# Assessing the Impact of Non‐Pharmaceutical Interventions During the COVID‐19 Pandemic on RSV Seasonality in Europe

**DOI:** 10.1111/irv.70066

**Published:** 2025-01-21

**Authors:** Susanne Heemskerk, Christos Baliatsas, Foekje Stelma, Harish Nair, John Paget, Peter Spreeuwenberg

**Affiliations:** ^1^ Nivel – Netherlands Institute for Health Services Research Utrecht The Netherlands; ^2^ Centre for Global Health, Usher Institute University of Edinburgh Edinburgh UK; ^3^ School of Public Health University of the Witwatersrand Johannesburg South Africa

**Keywords:** epidemiology, non‐pharmaceutical interventions, pandemic, public health, RSV, seasonality

## Abstract

**Background:**

During the COVID‐19 pandemic, atypical respiratory syncytial virus (RSV) circulation patterns emerged, with the occurrence of RSV activity outside the typical winter season. This study investigates the impact of COVID‐19 and associated non‐pharmaceutical interventions (NPIs) on RSV seasonality.

**Methods:**

The onset, offset and peak of RSV epidemics from 2018 to 2022 across 12 European countries were determined using the 3% positivity threshold method. A multilevel longitudinal logit regression model for proportions assessed the associations between five NPIs (school closures, mask use, workplace measures, public gathering restrictions and closure of public spaces) and RSV, utilising RSV surveillance data, two NPI databases (ECDC‐JRC and Oxford) and COVID‐19 surveillance data.

**Results:**

Before 2020, consistent RSV seasonality patterns were observed, but the seasonal increase of RSV‐positive cases in winter remained absent during the COVID‐19 pandemic (2020–2022). Analysis revealed inconsistent associations between individual NPIs and RSV. The associations differed depending on the data source used (ECDC‐JRC or Oxford), not only in magnitude but also in the direction of the coefficients. Public gathering restrictions and closure of public spaces exhibited significant negative associations with RSV incidence. However, this was only observed when using surveillance data for the entire epidemiological year and not when only examining weeks with increased RSV activity.

**Conclusions:**

This study highlights the need for standardised international data collection and procedures for infectious disease modelling, as varying NPI implementations, NPI registration and RSV surveillance across countries complicate the understanding of RSV dynamics during the pandemic. Caution is recommended when interpreting the effects of NPIs on RSV circulation.

## 1 Introduction

Respiratory syncytial virus (RSV) is a leading cause of respiratory infections in infants and young children and is increasingly recognised as a significant contributor to morbidity in older adults [1, 2, 3]. This viral respiratory infection has been extensively studied for its distinct seasonality, typically peaking during the winter months across Europe [3, 4]. However, the emergence of the COVID‐19 pandemic and implementation of various associated non‐pharmaceutical preventive measures disrupted this typical pattern, resulting in atypical RSV circulation patterns occurring outside the usual winter season timeframe in various countries [3, 5, 6, 7, 8, 9, 10]. Nevertheless, research on the relationship between the COVID‐19 pandemic and the observed alterations in RSV seasonality is still scarce.

The global response to the COVID‐19 pandemic involved various non‐pharmaceutical interventions (NPIs) that were implemented to control the spread of severe acute respiratory syndrome coronavirus 2 (SARS‐CoV‐2) [11]. In Europe, these measures were documented mainly in two publicly available databases: (1) the European Centre for Disease Prevention and Control (ECDC) in collaboration with the Joint Research Centre (JRC) Response Measures Database and (2) the Oxford COVID‐19 Government Response Tracker. NPIs ranged from national school closures and lockdowns to individual mask mandates [12, 13]. A recent comparative analysis of the NPI data in these two databases revealed notable differences in the recorded timing of the measures, even with seemingly similar definitions [14]. This emphasised the need for further exploration of the timing and implementation of various NPIs. Furthermore, given the remarkable change observed in RSV seasonality, it was hypothesised that the pandemic‐related NPIs had an impact on the seasonality of respiratory virus circulation, particularly on RSV.

Understanding changes in RSV seasonality is crucial for optimising public health responses, forecasting future epidemiological trends, and guiding timely interventions, such as vaccination or passive immunisation. This study aims to assess the impact of the COVID‐19 pandemic and the associated NPIs on RSV seasonality, as evidenced through surveillance data. RSV epidemics will be assessed annually for various countries. Additionally, exploratory analysis will determine the impact of NPIs on RSV during the epidemiological year and also focus on weeks with increased RSV activity (i.e., from onset to peak of an RSV epidemic). This timeframe is crucial as it is anticipated to show the most pronounced effects of NPIs on RSV transmission dynamics.

## 2 Methods

### 2.1 RSV Surveillance Data

RSV surveillance data were obtained from the ECDC Surveillance Atlas of Infectious Diseases, which collects data through The European Surveillance System (TESSy) [15]. TESSy includes sentinel surveillance in primary care and non‐sentinel surveillance in primary care and/or hospital facilities.

The dataset contains sentinel and non‐sentinel RSV data from 26 EU/EEA countries, including the number of RSV detections, total specimens tested and the percentage of sentinel and non‐sentinel surveillance specimens testing RSV‐positive. RSV data from countries were included if the following criteria were met: (1) ≥ 3 years of sufficient RSV detections available since 2018, (2) ≥ 50 RSV detections per epidemiological year for surveillance, which was defined as Week 27 through Week 26 of the next calendar year, (3) percentage of RSV‐positive tests or total number of specimens tested available (including non‐sentinel surveillance). Additionally, for the purpose of sensitivity analyses, seasons with substantial testing (> 500 tests) were included, even if RSV detections were below the ≥ 50 threshold. Data from the following countries were considered to be eligible based on these criteria: Bulgaria, Denmark, Estonia, France, Germany, Iceland, Ireland, Latvia, the Netherlands, Slovenia, Spain and Sweden. In these countries, non‐sentinel data were chosen for consistency, except for Germany and the Netherlands, where sentinel data were included because almost no non‐sentinel data was available.

### 2.2 NPI Databases

Two NPI databases were used: (1) the ECDC‐JRC Response Measures Database and (2) the Oxford COVID‐19 Government Response Tracker. The ECDC‐JRC NPI database is an archive of NPIs introduced by 30 countries in the EU/EEA from 1 January 2020 to 30 September 2022. Data entered into this database were collected by a dedicated team at ECDC, with measures implemented between January and March 2020 retrospectively gathered. This database contains measures organised at three levels to increase specificity and provide a detailed explanation on the aim of the measure [12]. These data were downloaded from the ECDC website. The Oxford NPI database collects government response measures in more than 185 countries worldwide from 1 January 2020 to 31 December 2022. Data entered into this database were collected by a team of over 1500 volunteers and were published in real time to understand variations in government responses. This database contains 23 indicators recorded on an ordinal scale that represents the level of strictness of the measure [13]. Oxford NPI data were downloaded directly from the ‘Our World in Data’ platform.

We selected five measures that we assumed were relevant to RSV transmission [3, 16, 17]: (1) closure of educational institutions, (2) protective mask use, (3) workplace measures, (4) public gathering restrictions and (5) closure of public spaces. Measures with the most similar definitions in both databases were included in our analyses. Detailed definitions of the measures can be found in Table S1. We synchronised the two databases and combined measures to ensure consistency in their definitions. More specifically, the strictest measures were selected from both databases. For the ECDC‐JRC NPI database, this means that measures were considered for inclusion when the status was mandatory, the implementation was full, the geographical level was national and the target group was the general population; the only exception was school closures, where the target groups consisted of students and teachers. This means that voluntary, partial and regional/local measures were not considered. For the Oxford NPI database, the highest stringency levels were selected for all five NPI measures included. Lastly, all included NPI variables in both databases were transformed into weekly measures.

### 2.3 COVID‐19 Surveillance Data

SARS‐CoV‐2 surveillance data were extracted from ‘Our World in Data’ for the selected European countries (see Section 2.1) [18]. We used the number of daily confirmed COVID‐19 cases and tests per country provided by the John Hopkins repository. The total number of cases and tests per week was calculated to match the weekly RSV surveillance data.

### 2.4 Statistical Analysis

We determined the onset and offset weeks of RSV epidemics in each country using the 3% positivity threshold method [19, 20]. The onset and offset weeks were defined as the first and last two consecutive weeks in which the percentage of sentinel or non‐sentinel surveillance specimens testing RSV‐positive exceeded 3%. This threshold was chosen to capture most RSV cases during the annual epidemics and because the percentage of RSV‐positive tests outside the epidemic seasons was typically < 3%. The peak week of the RSV epidemics was defined as the week with the highest percentage of RSV‐positive cases.

A multilevel longitudinal logit regression model for proportions was employed to assess the association between NPIs and RSV seasonality (see Supporting Information S1 for the model formula), taking into account the hierarchical structure of the data. In this model, the percentage of RSV‐positive tests (proportion) per week served as the dependent variable, with the total number of tests conducted in that week as the denominator. The multilevel structure incorporated ‘seasonal week (1‐52 or 53)’ as Level 1 units and ‘country’ as Level 2. For each year, a separate intercept was allowed in the model, allowing for the variability of RSV in a year (and, therefore, accounting for differences in RSV seasonality between countries). The average progression of seasonal weeks within an epidemiological year (starting in Week 27) was modelled as a polynomial fixed effect (to the 3rd order). Individual NPIs from either the ECDC‐JRC database or the Oxford database were included as independent variables (fixed effects). The model was estimated with MLwiN using PQL first‐order, RIGLS.

First, separate models were developed for individual NPIs for the ECDC‐JRC and Oxford databases. Next, the individual NPIs were added together to a multivariate model. To adjust for the potential confounding effect of COVID‐19 on RSV activity, weekly COVID‐19 positive test data were incorporated as fixed effects in the multilevel model. The rationale for this inclusion is based on evidence suggesting that high levels of COVID‐19 circulation can influence the patterns of other respiratory viruses, including RSV [21, 22]. To assess the robustness of our findings, we conducted analyses both with and without the inclusion of COVID‐19 data. To further explore the association between NPIs and RSV during the early stages of the annual epidemics, the weeks from the start to the peak of the RSV epidemic were incorporated as fixed effects into the model (varying between countries and years). This association reflects whether an NPI had a clear effect on RSV epidemics during weeks in which RSV activity was increasing. Two different scenarios were considered in the model analysis: (1) ≥ 50 RSV tests per week and (2) ≥ 50 RSV tests per week, excluding weeks with RSV rate = 0 and without considering seasonal fluctuations (no polynomial modelling of the ‘seasonal week’ variable).

The results of the investigated associations were presented as regression coefficients (β) with standard errors (SE). Two‐tailed tests were used to assess statistical significance, with a *p*‐value < 0.05 considered statistically significant. Descriptive analyses were conducted using Stata SE version 16.0 (StataCorp, 2019, College Station, TX), and multilevel analyses were performed using MlwiN (Centre for Multilevel Modelling, University of Bristol, Bristol, UK). The strength of the findings was evaluated based on statistical significance, the strength of the assessed associations, consistency in the observed associations regarding direction and strength (e.g., across different databases and scenarios), plausibility (considering existing literature) and relevance to public health or clinical practice.

## 3 Results

The univariate models showed associations between NPIs and RSV that were largely similar to those in the multilevel models, although some associations that were significant in the univariate model lost significance or changed direction. Overall, no clear patterns emerged in the associations between the individual NPIs and RSV in the univariate models, leading to the decision to not show these data and focus on the multilevel model, which incorporates COVID‐19 surveillance data (see Table 1). After adjusting for COVID‐19 in the multilevel models, the associations between NPIs and RSV remained similar (see Tables S3 and S4). Table 1 shows the variation of RSV between years and the variation between weeks within a year. It also shows the correlation between COVID‐19 surveillance data and RSV. Furthermore, it shows the associations of individual NPIs with RSV throughout the entire epidemiological year (defined as Week 27 through Week 26) and specifically during periods of increasing RSV circulation (from the onset to the peak of RSV epidemics).

**TABLE 1:** Detailed findings on the association (regression coefficients) between NPIs and RSV per database.

Parameter^a^	ECD‐JRC NPIs			Oxford NPIs		
	Estimate	SE	*p* value	Estimate	SE	*p* value
Year 2018	−1.44	0.60	0.02	−1.49	0.54	0.01
Year 2019	−1.32	0.60	0.03	−1.37	0.54	0.01
Year 2020	−2.41	0.60	< 0.01	−2.31	0.54	< 0.01
Year 2021	−1.06	0.60	0.08	−1.36	0.54	0.01
Year 2022	−2.73	0.60	< 0.01	−2.96	0.54	< 0.01
Week	−0.11	0.00	< 0.01	−0.11	0.00	< 0.01
Week 2	0.00	0.00	< 0.01	0.00	0.00	< 0.01
Week 3	0.00	0.00	< 0.01	0.00	0.00	< 0.01
RSV season	0.53	0.01	< 0.01	0.46	0.01	< 0.01
COVID‐19	−0.03	0.00	< 0.01	−0.01	0.00	< 0.01
School closures	1.28	0.03	< 0.01	0.01	0.04	0.74
Protective mask use	−0.23	0.01	< 0.01	0.49	0.01	< 0.01
Workplace measures	1.35	0.03	< 0.01	−0.05	0.04	0.24
Public gathering restrictions	−0.57	0.03	< 0.01	−0.88	0.02	< 0.01
Closure of public spaces	−2.19	0.03	< 0.01	−0.56	0.02	< 0.01
I_school closures	0.05	0.44	0.91	0.25	0.12	0.03
I_protective mask use	0.19	0.05	< 0.01	0.41	0.02	< 0.01
I_workplace measures	−0.92	0.42	0.03	0.31	0.05	< 0.01
I_public gathering restrictions	0.44	0.05	< 0.01	0.45	0.03	< 0.01
I_ closure of public spaces	1.10	0.42	0.01	−0.33	0.04	< 0.01

^a^ Year describes the variation between years for countries; week describes the variation between weeks within a year (polynomial); measure describes the interaction between individual NPIs and RSV during the epidemiological year (defined as Week 27 through Week 26); I_measure describes the interaction between NPIs and RSV during weeks in which RSV activity is increasing (from the onset to peak of RSV epidemics). This model describes the outcome of Scenario 1, that is, ≥ 50 RSV tests per week.

Abbreviations: NPIs, non‐pharmaceutical interventions; RSV, respiratory syncytial virus; SE, standard error.

### 3.1 Description of RSV Seasonality

**FIGURE 1:**
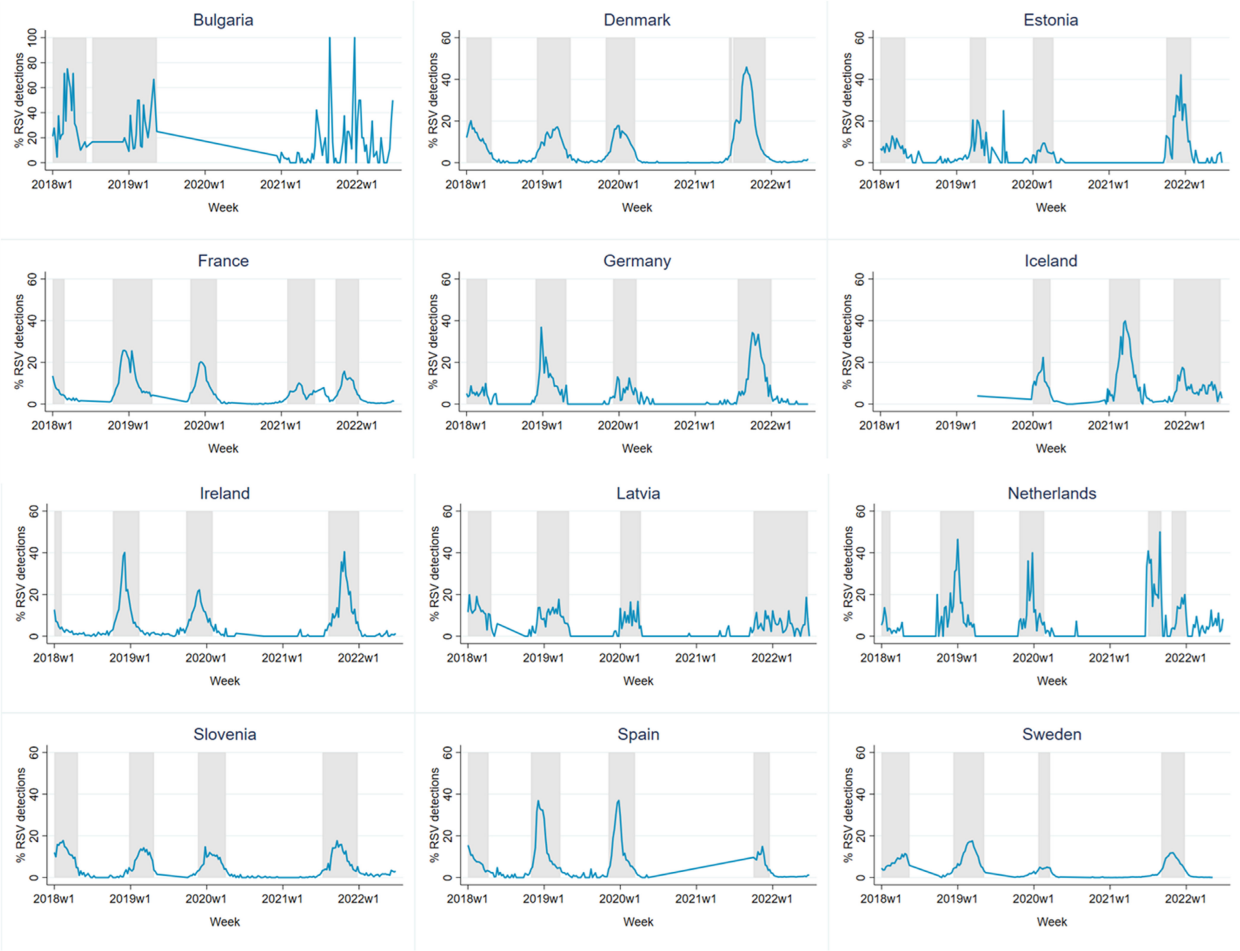
Percentage of RSV‐positive detections of included countries; grey areas indicate RSV epidemics. RSV, respiratory syncytial virus.

The seasonality of RSV across different countries revealed a consistent pattern—see Figure 1 for an overview of the percentage of RSV‐positive detections per country and see Table S2 for the onset, offset and peak of RSV epidemics per country for 2018–2022. More specifically, RSV epidemics typically occurred from late fall to early spring, with peaks observed from November to March, although slight variations in the timing of RSV epidemics and peaks from year to year were observed. Differences in RSV seasonality before and during the COVID‐19 pandemic were mainly observed by the low rate and seasonal delay of RSV‐positive cases during the 2020–2022 seasons. Specifically in 2020–2021, where almost no RSV was detected in Denmark, Estonia, Germany, Ireland, Latvia, the Netherlands, Slovenia, Spain and Sweden. Additionally, in the Netherlands, two epidemic periods were observed during the 2021–2022 season (Table S2).

### 3.2 Association Between RSV and NPIs Based on the ECDC‐JRC Data

The association between NPIs and RSV per database is provided in Table 1. Examining RSV surveillance data for the epidemiological year in relation to ECDC‐JRC revealed significant negative associations between RSV presence and specific NPIs, namely, protective mask use (β = −0.23, SE = 0.01, *p* < 0.01), public gathering restrictions (β = −0.57, SE = 0.03, *p* < 0.01) and closure of public spaces (β = −2.19, SE = 0.03, *p* < 0.01).

The negative estimates for these NPIs suggest a protective effect of the NPI studied, indicating a decrease in RSV transmission associated with the implementation of these interventions. However, when focusing on weeks with increased RSV activity (from the onset to peak of RSV epidemics), where stronger effects are expected than when using year‐round data, only workplace measures (β = −0.92, SE = 0.42, *p* = 0.03) exhibited a significant negative association. Conversely, analyses yielded significant positive associations with RSV for protective mask use, public gathering restrictions and closure of public spaces.

### 3.3 Association Between RSV and NPIs Based on the Oxford Data

When using RSV surveillance data for the whole epidemiological year and NPIs from the Oxford database, public gathering restrictions (β = −0.88, SE = 0.02, *p* < 0.01) and the closure of public spaces (β = −0.56, SE = 0.02, *p* < 0.01) exhibited significant negative associations with RSV, indicating a potential decrease in RSV transmission due to the implementation of these measures (Table 1). On the other hand, the use of protective masks was significantly and positively associated with RSV (β = 0.49, SE = 0.01, *p* < 0.01), suggesting an increase in RSV transmission.

When the analyses focused solely on the weeks with increased RSV activity, significant positive associations were found for most of the measures, including school closures, protective mask use, workplace measures and public gathering restrictions. The only exception was the closure of public spaces, which was negatively associated with RSV (β = −0.33, SE = 0.04, *p* < 0.01) during these weeks.

### 3.4 Comparison of ECD‐JRC and Oxford Model Outcomes

When comparing the outcomes of the model using the ECD‐JRC and Oxford databases, notable discrepancies were observed in the associations between the individual NPIs and RSV (Table 1). These differences did not only concern the magnitude of associations but also the direction of the coefficients (whether positive or negative), ultimately leading to contradictory results.

#### 3.4.1 Consistent Findings Between Databases

In the analysis spanning the entire epidemiological year, only two measures—public gathering restrictions and closure of public spaces—demonstrated significant negative associations with RSV in both databases. When analyses were restricted to weeks with increased RSV activity in the model, no negative associations were observed between RSV and NPIs corresponding between the databases. However, protective mask use and public gathering restrictions were positively associated with RSV in both datasets.

Notably, public gathering restrictions and closure of public spaces were the only corresponding measures between Scenario 1 (≥ 50 RSV tests per week) and Scenario 2 (≥ 50 RSV tests per week, excluding weeks where the RSV rate equals zero and not considering seasonal fluctuations) for both databases, demonstrating a significant negative association with RSV (Table S5). However, this effect was only observed when using year‐round surveillance data and was, therefore, not apparent when only examining weeks with increasing RSV activity. No associations between COVID‐19 surveillance data and RSV were observed in both databases (Table 1).

#### 3.4.2 Contradictory Findings Between Databases

Interestingly, protective mask use showed a significant negative impact on RSV in the ECD‐JRC database, while demonstrating a positive association in the Oxford database when using year‐round surveillance data. When only looking at weeks with increasing RSV activity, workplace measures showed a significant negative association with RSV using the ECD‐JRC database but a positive association when using the Oxford database. Conversely, the closure of public spaces revealed the opposite effect in the two databases. When adopting a stricter model scenario (Scenario 2), inconsistencies in the associations between individual NPIs and RSV were evident across the two databases (Table S5). This scenario also showed conflicting results with Scenario 1, revealing distinct associations for NPIs.

## 4 Discussion

This study aimed to assess the association between NPIs and RSV seasonality using publicly accessible surveillance data and two different NPI databases. Our study revealed conflicting findings regarding the impact of individual NPIs on RSV activity. Substantial disparities in associations emerged when using data from the ECDC‐JRC and Oxford NPI databases, as indicated by variations in the direction and magnitude of the coefficients. In contrast with existing literature [23, 24, 25, 26], our study highlights that public gathering restrictions and the closure of public spaces were the only measures that exhibited significant negative associations with RSV across both databases, suggesting a potential reduction in RSV due to the implementation of these measures. However, these associations were not observed when exclusively examining periods of increased RSV activity. These results underscore the challenges in drawing definitive conclusions regarding the relationship between NPIs and RSV.

Surprisingly, the majority of associations between individual NPIs and RSV deviated from the expected direction (negative or zero), suggesting an increase in RSV activity when NPIs were implemented (for both the univariate and multivariate models). These findings are in contrast to the existing literature. In a recent study where investigators also used the Oxford COVID‐19 Government Response Tracker for the investigated NPIs, reopening of schools and relaxation of stay‐at‐home measures were linked to increased RSV activity [23]. However, their study investigated whether NPIs contributed to a delayed onset of the seasonal RSV epidemic, and their analysis confirmed this effect. Additionally, that study took into account a 10‐week time lag between NPIs and the start of an increase in RSV‐positive cases. Another study reported similar results, suggesting that the reopening of schools correlated with a heightened risk of RSV resurgence [24]. However, the results of the latter study may not be directly comparable to our analyses due to a time‐dependent analysis and a different method for assessing different NPIs and other exposures.

A critical aspect to consider when interpreting our findings is the quality of the data utilised. A previous comparative analysis highlighted disparities in the timing of interventions between the ECDC‐JRC and Oxford NPI databases [14]. It is, therefore, likely that the databases have collected different observations resulting in different associations between the individual NPIs and RSV epidemics in this study. The differences between the ECDC‐JRC and Oxford NPI databases could be explained by discrepancies in the definitions of measures or variations in the registration of NPIs. Additionally, to achieve comparable definitions of measures across both databases, we standardised the granularity of the raw data during the construction of measures, potentially influencing the results. Our analysis does not provide sufficient evidence to determine which database performs better. Furthermore, the variability of NPIs within and between countries could also account for differences in model outcomes. Some countries, for example, Spain, implemented not only national measures but also regional measures. Adherence to measures is also of importance both within individual countries and between countries, yet adherence to NPIs was not considered in our analysis.

Similarly, RSV surveillance data exhibited variability not only between countries but also between different years within the same country, influenced by differences in testing and surveillance systems across Europe [27, 28]. The 3% positivity threshold method used in this study effectively captured peaks during RSV epidemics; see Figure 1. However, in instances where RSV circulates continuously over an extended period, such as observed in Bulgaria during 2020–2022 and in the Netherlands during 2021–2022, this method may not be as suitable for identifying epidemics. RSV surveillance data from TESSy, originally established to monitor influenza, were used for this study. Other studies used different RSV surveillance outputs [23]. Using surveillance data from Tessy may not be perfectly suited for measuring RSV. The European Influenza Surveillance Network (EISN) reports RSV data to TESSy through both acute respiratory infection (ARI) and influenza‐like illness (ILI) sentinel and non‐sentinel surveillance systems (1). The ILI case definition is not optimal for capturing RSV because this can lead to under‐detection of RSV in countries employing this case definition [29]. Additionally, non‐sentinel data were used for most countries, except for Germany and the Netherlands. The majority of RSV‐positive cases reported to TESSy come from non‐sentinel surveillance, collecting data based on diagnostic needs. Non‐sentinel surveillance is likely to have been impacted by the COVID‐19 pandemic, resulting in underreporting of RSV‐positive cases. At the beginning of the COVID‐19 pandemic, SARS‐CoV‐2 surveillance was also mainly non‐sentinel, leading to an underestimation of COVID‐19‐positive cases during the spring and summer of 2020 [30]. However, because COVID‐19 was only used as a control variable, this is not expected to have influenced our results.

The main strength of this study is the use of two NPI databases involving many European countries. This enables a comprehensive assessment of the association between NPIs and RSV. However, several limitations should be acknowledged. First is the reliance on publicly available databases, which may not always provide an optimal quality of the data. The clear differences between the NPI databases and the variability between and within countries of the RSV surveillance data underscore the necessity for caution when interpreting the results. Furthermore, the study did not account for potential confounding factors, such as social adherence to measures and weather conditions (e.g., temperature) [31]. Incorporating these variables can enhance the accuracy of future models and provide a more nuanced understanding of the relationship between NPIs and the occurrence of seasonal RSV epidemics. Additionally, exploring country‐specific associations between NPIs and RSV may offer more accurate insights into the various associations.

The above‐mentioned shortcomings lead to the following recommendations for future studies and RSV surveillance: First, European RSV surveillance should be standardised, ideally to match international influenza surveillance. This also includes the standardisation of a uniform case definition for RSV [32]. Moreover, improving European consistency and uniformity when registering NPIs, including standardised definitions across databases, will improve data quality and facilitate more robust analyses. Overall, the conflicting findings and methodological limitations highlighted in this study underscore the complexity of the impact of the COVID‐19 pandemic and associated NPIs on RSV seasonality.

## 5 Conclusions

In conclusion, the present study highlights the complexity of assessing the association between NPIs during the recent COVID‐19 pandemic and RSV seasonality. While some NPIs exhibited negative associations with RSV epidemic occurrence, suggesting a potential protective effect, conflicting findings were also observed, questioning the quality of the databases used for this analysis and challenging existing literature. Methodological limitations were characterised by important differences between the ECDC‐JRC and Oxford NPI databases, probably implementation differences between countries of NPIs, and by high variability in RSV surveillance data between countries. This emphasises the necessity for caution when interpreting these results. This study also underscores the importance of rigorous surveillance, also during pandemics, leading to improved data quality on the incidence and prevalence of infection. This will improve our ability to model infectious diseases like RSV and be able to improve our understanding of RSV dynamics during pandemics.

## Author Contributions

**Susanne Heemskerk:** conceptualization, data curation, writing – original draft, writing – review and editing. **Christos Baliatsas:** supervision, writing – review and editing. **Foekje Stelma:** supervision, project administration. **Harish Nair:** conceptualization, writing – review and editing. **John Paget:** conceptualization, supervision, project administration, funding acquisition. **Peter Spreeuwenberg:** conceptualization, data curation, formal analysis, supervision, writing – review and editing.

## Ethics Statement

The study does not fall within the scope of the Medical Research Involving Human Subjects Act and therefore does not require ethical approval.

## Consent

Considering the study design and the fact that analyses were based on publicly available data, written informed consent was not applicable.

## Conflicts of Interest

JP declared that Nivel has received unrestricted grants from the World Health Organization, Sanofi and the Foundation for Influenza Epidemiology outside the submitted work. HN reports grants from the World Health Organization, the National Institute for Health Research, Pfizer and Icosavax and personal fees from the Bill & Melinda Gates Foundation, Pfizer, GSK, Merck, AbbVie, Janssen, Icosavax, Sanofi and Novavax outside the submitted work.

### Peer Review

The peer review history for this article is available at https://www.webofscience.com/api/gateway/wos/peer‐review/10.1111/irv.70066.

## Supporting information


**Table S1.** Detailed definition of measures for ECDC‐JRC Response Measures Database and Oxford COVID‐19 Government Response Tracker.
**Supporting Information S1.** Statistical model formula, logistic model for proportions.
**Table S2.** Onset, peak and offset of the RSV epidemics and number of RSV detections per season of included countries.
**Table S3.** Comparison of the detailed findings on the association (regression coefficients) between ECDC‐JRC NPIs and RSV, with and without the influence of COVID‐19.
**Table S4.** Comparison of the detailed findings on the association (regression coefficients) between Oxford NPIs and RSV, with and without the influence of COVID‐19.
**Table S5.** Detailed findings on the association (regression coefficients) between NPIs and RSV, per database*.

## Data Availability

The data that support the findings of this study are publicly available, as well as available from the authors upon reasonable request.
