# Supplementary material for: Assessing the Impact of Non‐Pharmaceutical Interventions During the COVID‐19 Pandemic on RSV Seasonality in Europe
Source: Influenza Other Respir Viruses. 2025 Jan 21;19(1):e70066. doi: 10.1111/irv.70066 (PMC11750802; doi:10.1111/irv.70066)
Supplement: Supplementary file 1 — Table S1. Detailed definition of measures for ECDC‐JRC Response Measures Database and Oxford COVID‐19 Government Response Tracker. Supporting Information S1. Statistical model formula, logistic model for proportions. Table S2. Onset, peak and offset of the RSV epidemics and number of RSV detections per season of included countries. Table S3. Comparison of the detailed findings on the association (regression coefficients) between ECDC‐JRC NPIs and RSV, with and without the influence of COVID‐19. Table S4. Comparison of the detailed findings on the association (regression coefficients) between Oxford NPIs and RSV, with and without the influence of COVID‐19. Table S5. Detailed findings on the association (regression coefficients) between NPIs and RSV, per database*. [file IRV-19-e70066-s001.docx]

**Supporting Information**

**Table of contents**

[Supplementary Table 1. Detailed definition of measures for ECDC-JRC Response Measures Database and Oxford COVID-19 Government Response Tracker 2](#_Toc182386985)

[Supplementary material 1. Statistical model formula, logistic model for proportions 4](#_Toc182386986)

[Supplementary Table 2. Onset, peak and offset of the RSV epidemics and number of RSV detections per season of included countries 5](#_Toc182386987)

[Supplementary Table 3. Comparison of the detailed findings on the association (regression coefficients) between ECDC-JRC NPIs and RSV, with and without the influence of COVID-19 7](#_Toc182386988)

[Supplementary Table 4. Comparison of the detailed findings on the association (regression coefficients) between Oxford NPIs and RSV, with and without the influence of COVID-19 8](#_Toc182386989)

[Supplementary Table 5. Detailed findings on the association (regression coefficients) between NPIs and RSV, per database* 9](#_Toc182386990)

Supplementary Table 1. Detailed definition of measures for ECDC-JRC Response Measures Database and Oxford COVID-19 Government Response Tracker

| **Measure** | **ECDC-JRC Response Measures Database** | **Oxford COVID-19 Government Response Tracker** |
| --- | --- | --- |
| Closure of educational institutions | Closure of educational institutions   - Daycare nursery - Primary school | C1. Records closings of schools and universities^†^   - Require closing all levels (stringency level 3) |
| Protective mask use | Protective mask use community   - All public spaces - Closed public spaces (e.g., transportation facilities, supermarkets or working environments) | H6. Records policies on the use of facial coverings outside the home   - Required in all shared/public spaces outside the home with other people present or all situations when social distancing not possible (level 3) - Required outside the home at all times regardless of location or presence of other people (level 4) |
| Workplace measures | Workplace measures   - Closure of workplaces - Teleworking (specific recommendation of teleworking from home) | C2. Records closings of workplaces   - Require closing (or work from home) for all-but-essential workplaces (e.g., grocery stores, doctors) (level 3) |
| Public gathering restrictions | Public gathering restrictions: Any measure or legislation which limits participation or attendance to a public event   - Indoor over 50 (between 31 and 50 participants) and 100 (between 51 and 100 participants) - Outdoor over 50 (between 31 and 50 participants) and 100 (between 51 and 100 participants) - Ban on all events | C4. Records the cut-off size for limits on gatherings   - Restrictions on gatherings between 11-100 people (level 3) - Restrictions on gatherings of 10 people or less (level 4) |
| Closure of public spaces | Closure of public spaces   - Entertainment venues (e.g., arena, concert halls, theatre) - Gym, sports centres - Hotels and other accommodation - Non-essential shops - Place of worship - Restaurants or cafes - Other public spaces | C3. Records cancelling public events   - Require cancelling (level 2)^‡^ |

† C1 reports closures of both schools and universities. It does not report closures of childcare, nurseries, language courses, and driving schools, which are instead recorded as workplaces under C2.

‡ When private gatherings of only 10 people or less are permitted (i.e., C4=4), this restriction would prevent public events from taking place, so we also report C3=2 (public events are required to be cancelled), unless there is a specific policy in place permitting public gatherings to go ahead.

Supplementary material 1. Statistical model formula, logistic model for proportions

Y_i,j_ = β_0,year_ + β_(i,j)_rsv + β_(i,j)_week + β_(i,j)_npi + β_(i,j)_npiXrsv(increase) + β_(i,j)_covid + µ_(j)_ + є_(i,j)_

Y_i,j_ : outcome measure, number of positive tests per 1000 tests per week.

I: week=(year-2017*100)+week number

J: country

β_0,year_ : intercept for every year

β_(i,j)_week : third order polynomial for week (linear, quadratic, cubic term)

β_(i,j)_rsv : indicator if RSV is active in that week in a country

β_(i,j)_npi : indicator if a NPI is active in that week in a country, separate indicator

for every NPI

β_(i,j)_npiXrsv(increase): indicator if a NPI is active during a week in which the

RSV-epidemic is increasing in a country, separate indicator for

every NPI

β_(i,j)_covid : proportion of positive covid test in a week in a country

µ_(j)_ : between country variance

є_(i,j)_ : error variance, for a logistic model, binomial distribution, with a logit

link function, constrained to 1, estimation procedure: RIGLS , PQL second

order

Supplementary Table 2. Onset, peak and offset of the RSV epidemics and number of RSV detections per season of included countries

| **Country,**  **season** | **Start, week** | **Peak, week** | **End,**  **week** | **RSV+ cases**^†^ | **Total specimens tested** | **Data source** |
| --- | --- | --- | --- | --- | --- | --- |
| Bulgaria |  |  |  |  |  | Non-sentinel |
| 2017-2018 | 34 - 2017 | 11 - 2018 | 24 - 2018 | 90 | 446 |  |
| 2018-2019 | 28 - 2018 | 18 - 2019 | 20 - 2019 | 107 | 554 |  |
| 2019-2020 | - | - | - | - | - |  |
| 2020-2021 | - | - | - | 78 | 749 |  |
| 2021-2022 | - | - | - | 54 | 334 |  |
| Denmark |  |  |  |  |  | Non-sentinel |
| 2017-2018 | 46 - 2017 | 4 - 2018 | 18 - 2018 | 4504 | 42860 |  |
| 2018-2019 | 49 - 2018 | 11 - 2019 | 20 - 2019 | 4184 | 42642 |  |
| 2019-2020 | 44 - 2019 | 52 - 2019 | 12 - 2020 | 4471 | 56606 |  |
| 2020-2021 |  |  |  | 127 | 25676 |  |
| 2021-2022 | 24 - 2021 | 36 - 2021 | 49 - 2021 | 10961 | 121418 |  |
| Estonia |  |  |  |  |  | Non-sentinel |
| 2017-2018 | 50 - 2017 | 9 - 2018 | 18 - 2018 | 571 | 8266 |  |
| 2018-2019 | 10 - 2019 | 15 - 2019 | 21 - 2019 | 299 | 8027 |  |
| 2019-2020 | 1 - 2020 | 8 - 2020 | 15 - 2020 | 260 | 5427 |  |
| 2020-2021 | - | - | - | 0* | 3031 |  |
| 2021-2022 | 40 - 2021 | 50 - 2021 | 5 -2022 | 227 | 1976 |  |
| France |  |  |  |  |  | Non-sentinel |
| 2017-2018 | 42 - 2017 | 47 - 2017 | 9 - 2018 | 11524 | 121938 |  |
| 2018-2019 | 42 - 2018 | 50 - 2018 | 17 - 2019 | 14346 | 95902 |  |
| 2019-2020 | 43 - 2019 | 50 - 2019 | 9 - 2020 | 11082 | 163805 |  |
| 2020-2021 | 5 - 2021 | 13 - 2021 | 24 - 2021 | 5984 | 140983 |  |
| 2021-2022 | 38 - 2021 | 44 - 2021 | 2 - 2022 | 13630 | 344604 |  |
| Germany |  |  |  |  |  | Sentinel |
| 2017-2018 | 51 - 2017 | 52 - 2017 | 15 - 2018 | 254 | 6239 |  |
| 2018-2019 | 48 - 2018 | 52 - 2018 | 17 - 2019 | 367 | 4205 |  |
| 2019-2020 | 49 - 2019 | 8 - 2020 | 13 - 2020 | 201 | 4364 |  |
| 2020-2021 | - | - | - | 10* | 5849 |  |
| 2021-2022 | 30 - 2021 | 40 - 2021 | 1 - 2022 | 805 | 7570 |  |
| Iceland |  |  |  |  |  | Non-sentinel |
| 2019-2020 | 1 - 2020 | 8 - 2020 | 13 - 2020 | 270 | 3526 |  |
| 2020-2021 | 1 - 2021 | 12 - 2021 | 22 - 2021 | 529 | 5285 |  |
| 2021-2022 | 45 - 2021 | 51 - 2021 | 25 - 2022 | 552 | 8902 |  |
| Ireland |  |  |  |  |  | Non-sentinel |
| 2017-2018 | 40 - 2017 | 49 - 2017 | 6 - 2018 | 1543 | 19168 |  |
| 2018-2019 | 41 - 2018 | 49 - 2018 | 7 - 2019 | 1521 | 19981 |  |
| 2019-2020 | 39 - 2019 | 48 - 2019 | 5 - 2020 | 1445 | 17913 |  |
| 2020-2021 | - | - | - | 5* | 4120 |  |
| 2021-2022 | 32 - 2021 | 43 - 2021 | 1 -2022 | 825 | 8857 |  |
| Latvia |  |  |  |  |  | Non-sentinel |
| 2017-2018 | 40 - 2017 | 2 - 2018 | 17 - 2018 | 519 | 5052 |  |
| 2018-2019 | 48 - 2018 | 11 - 2019 | 18 - 2019 | 263 | 2924 |  |
| 2019-2020 | 1 - 2020 | 13 - 2020 | 15 - 2020 | 110 | 1825 |  |
| 2020-2021 | - | - | - | 4* | 1706 |  |
| 2021-2022 | 40 - 2021 | 48 - 2021 | 25 - 2022 | 114 | 2353 |  |
| Netherlands |  |  |  |  |  | Sentinel |
| 2017-2018 | 46 - 2017 | 51 - 2017 | 7 - 2018 | 75 | 1272 |  |
| 2018-2019 | 41 - 2018 | 1 - 2019 | 12 - 2019 | 105 | 955 |  |
| 2019-2020 | 43 - 2019 | 52 - 2019 | 8 - 2020 | 93 | 1689 |  |
| 2020-2021 | - | - | - | 5* | 630 |  |
| 2021-2022 | 27 - 2021 | 35 - 2021 | 36 - 2021 | 108 | 1520 |  |
| 2021-2022 | 43 - 2021 | 52 - 2021 | 1 - 2022 | 108 | 1520 |  |
| Slovenia |  |  |  |  |  | Non-sentinel |
| 2017-2018 | 50 - 2017 | 7 - 2018 | 17 - 2018 | 1537 | 17360 |  |
| 2018-2019 | 52 - 2018 | 10 - 2019 | 17 - 2019 | 1228 | 17384 |  |
| 2019-2020 | 47 - 2019 | 52 - 2019 | 14 - 2020 | 1369 | 18377 |  |
| 2020-2021 | - | - | - | 31* | 18686 |  |
| 2021-2022 | 28 - 2021 | 38 - 2021 | 52 - 2021 | 1811 | 37013 |  |
| Spain |  |  |  |  |  | Non-sentinel |
| 2017-2018 | 42 - 2017 | 49 - 2017 | 15 - 2018 | 3967 | 35381 |  |
| 2018-2019 | 44 - 2018 | 49 - 2018 | 12 - 2019 | 4346 | 39503 |  |
| 2019-2020 | 45 - 2019 | 52 - 2019 | 11 - 2020 | 4585 | 39095 |  |
| 2020-2021 | - | - | - | - | - |  |
| 2021-2022 | 40 - 2021 | 46 - 2021 | 51 - 2021 | 4949 | 286417 |  |
| Sweden |  |  |  |  |  | Non-sentinel |
| 2017-2018 | 51 - 2017 | 17 - 2018 | 20 - 2018 | 4528 | 73795 |  |
| 2018-2019 | 50 - 2018 | 11 - 2019 | 19 - 2019 | 7312 | 75623 |  |
| 2019-2020 | 4 - 2020 | 10 - 2020 | 12 - 2020 | 1987 | 72364 |  |
| 2020-2021 | - | - | - | 308 | 209830 |  |
| 2021-2022 | 36 - 2021 | 44 - 2021 | 52 - 2021 | 16042 | 455049 |  |

†Empty cells have <50 RSV detections per season, RSV detections with * also have <50 RSV detections per season but is included in the table because a lot of testing was performed (>500 tests).

The following countries did not have sufficient data: Belgium, Croatia, Cyprus, Czechia, Finland, Greece, Hungary, Italy, Lithuania, Luxembourg, Malta, Poland, Portugal, Romania, and Slovakia.

Supplementary Table 3. Comparison of the detailed findings on the association (regression coefficients) between ECDC-JRC NPIs and RSV, with and without the influence of COVID-19

|  | **No COVID-19 in the model** | | | **COVID-19 in the model** | | |
| --- | --- | --- | --- | --- | --- | --- |
| **Parameter^†^** | **Estimate** | **SE** | **P value** | **Estimate** | **SE** | **P value** |
| Year 2018 | -1.55 | 0.53 | <0.01 | -1.44 | 0.60 | 0.02 |
| Year 2019 | -1.43 | 0.53 | 0.01 | -1.32 | 0.60 | 0.03 |
| Year 2020 | -2.72 | 0.53 | <0.01 | -2.41 | 0.60 | <0.01 |
| Year 2021 | -1.33 | 0.53 | 0.01 | -1.06 | 0.60 | 0.08 |
| Year 2022 | -3.49 | 0.53 | <0.01 | -2.73 | 0.60 | <0.01 |
| Week | -0.11 | 0.00 | <0.01 | -0.11 | 0.00 | <0.01 |
| Week^2^ | 0.00 | 0.00 | <0.01 | 0.00 | 0.00 | <0.01 |
| Week^3^ | 0.00 | 0.00 | <0.01 | 0.00 | 0.00 | <0.01 |
| RSV season | 0.54 | 0.01 | <0.01 | 0.53 | 0.01 | <0.01 |
| COVID-19 | - | - | - | -0.03 | 0.00 | <0.01 |
| School closures | 1.15 | 0.03 | <0.01 | 1.28 | 0.03 | <0.01 |
| Protective mask use | -0.28 | 0.01 | <0.01 | -0.23 | 0.01 | <0.01 |
| Workplace measures | 1.48 | 0.03 | <0.01 | 1.35 | 0.03 | <0.01 |
| Public gathering restrictions | -0.72 | 0.03 | <0.01 | -0.57 | 0.03 | <0.01 |
| Closure of public spaces | -2.10 | 0.03 | <0.01 | -2.19 | 0.03 | <0.01 |
| I_school closures | 0.17 | 0.44 | 0.7 | 0.05 | 0.44 | 0.91 |
| I_protective mask use | 0.26 | 0.05 | <0.01 | 0.19 | 0.05 | <0.01 |
| I_workplace measures | -0.97 | 0.42 | 0.02 | -0.92 | 0.42 | 0.03 |
| I_public gathering restrictions | 0.42 | 0.05 | <0.01 | 0.44 | 0.05 | <0.01 |
| I_ closure of public spaces | 1.06 | 0.42 | 0.01 | 1.10 | 0.42 | 0.01 |

† Year describes the variation between years for countries; week describes the variation between weeks within a year (polynomial); measure describes the interaction between individual NPIs and RSV during the epidemiological year (defined as week 27 through week 26); I_measure describes the interaction between NPIs and RSV during weeks in which RSV activity is increasing (from the onset to peak of RSV epidemics). This model describes the outcome of scenario 1, i.e., ≥50 RSV tests per week.

Abbreviations: NPIs, non-pharmaceutical interventions; SE, standard error; RSV, respiratory syncytial virus

Supplementary Table 4. Comparison of the detailed findings on the association (regression coefficients) between Oxford NPIs and RSV, with and without the influence of COVID-19

|  | **No COVID-19 in the model** | | | **COVID-19 in the model** | | |
| --- | --- | --- | --- | --- | --- | --- |
| **Parameter^†^** | **Estimate** | **SE** | **P value** | **Estimate** | **SE** | **P value** |
| Year 2018 | -1.55 | 0.50 | <0.01 | -1.49 | 0.54 | 0.01 |
| Year 2019 | -1.43 | 0.50 | <0.01 | -1.37 | 0.54 | 0.01 |
| Year 2020 | -2.41 | 0.50 | <0.01 | -2.31 | 0.54 | <0.01 |
| Year 2021 | -1.48 | 0.50 | <0.01 | -1.36 | 0.54 | 0.01 |
| Year 2022 | -3.38 | 0.50 | <0.01 | -2.96 | 0.54 | <0.01 |
| Week | -0.11 | 0.00 | <0.01 | -0.11 | 0.00 | <0.01 |
| Week^2^ | 0.00 | 0.00 | <0.01 | 0.00 | 0.00 | <0.01 |
| Week^3^ | 0.00 | 0.00 | <0.01 | 0.00 | 0.00 | <0.01 |
| RSV season | 0.47 | 0.01 | <0.01 | 0.46 | 0.01 | <0.01 |
| COVID-19 | - | - | - | -0.01 | 0.00 | <0.01 |
| School closures | -0.03 | 0.04 | 0.54 | 0.01 | 0.04 | 0.74 |
| Protective mask use | 0.45 | 0.01 | <0.01 | 0.49 | 0.01 | <0.01 |
| Workplace measures | 0.03 | 0.04 | 0.54 | -0.05 | 0.04 | 0.24 |
| Public gathering restrictions | -0.93 | 0.01 | <0.01 | -0.88 | 0.02 | <0.01 |
| Closure of public spaces | -0.59 | 0.02 | <0.01 | -0.56 | 0.02 | <0.01 |
| I_school closures | 0.26 | 0.12 | 0.03 | 0.25 | 0.12 | 0.03 |
| I_protective mask use | 0.43 | 0.02 | <0.01 | 0.41 | 0.02 | <0.01 |
| I_workplace measures | 0.19 | 0.05 | <0.01 | 0.31 | 0.05 | <0.01 |
| I_public gathering restrictions | 0.49 | 0.03 | <0.01 | 0.45 | 0.03 | <0.01 |
| I_ closure of public spaces | -0.29 | 0.04 | <0.01 | -0.33 | 0.04 | <0.01 |

† Year describes the variation between years for countries; week describes the variation between weeks within a year (polynomial); measure describes the interaction between individual NPIs and RSV during the epidemiological year (defined as week 27 through week 26); I_measure describes the interaction between NPIs and RSV during weeks in which RSV activity is increasing (from the onset to peak of RSV epidemics). This model describes the outcome of scenario 1, i.e., ≥50 RSV tests per week.

Abbreviations: NPIs, non-pharmaceutical interventions; SE, standard error; RSV, respiratory syncytial virus

Supplementary Table 5. Detailed findings on the association (regression coefficients) between NPIs and RSV, per database*

|  | **ECD-JRC NPIs** | | | **Oxford NPIs** | | |
| --- | --- | --- | --- | --- | --- | --- |
| **Parameter**** | **Estimate** | **SE** | **P value** | **Estimate** | **SE** | **P value** |
| Year 2018 | -2.08 | 0.53 | <0.01 | -2.08 | 0.49 | <0.01 |
| Year 2019 | -1.93 | 0.53 | <0.01 | -1.94 | 0.49 | <0.01 |
| Year 2020 | -2.94 | 0.53 | <0.01 | -2.82 | 0.49 | <0.01 |
| Year 2021 | -1.90 | 0.53 | <0.01 | -1.93 | 0.49 | <0.01 |
| Year 2022 | -3.97 | 0.53 | <0.01 | -3.96 | 0.49 | <0.01 |
| RSV season | 0.53 | 0.01 | <0.01 | 0.52 | 0.01 | <0.01 |
| COVID-19 | -0.01 | 0.00 | <0.01 | 0.00 | 0.00 | <0.01 |
| School closures | 1.54 | 0.04 | <0.01 | 0.36 | 0.05 | <0.01 |
| Protective mask use | -0.06 | 0.01 | <0.01 | 0.50 | 0.01 | <0.01 |
| Workplace measures | 0.66 | 0.03 | <0.01 | 0.09 | 0.04 | 0.03 |
| Public gathering restrictions | -0.50 | 0.03 | <0.01 | -1.03 | 0.01 | <0.01 |
| Closure of public spaces | -2.08 | 0.04 | <0.01 | -0.45 | 0.02 | <0.01 |
| I_school closures | -0.79 | 0.45 | 0.08 | -0.55 | 0.12 | <0.01 |
| I_protective mask use | 0.07 | 0.05 | 0.14 | 0.18 | 0.02 | <0.01 |
| I_workplace measures | -0.35 | 0.42 | 0.41 | -0.05 | 0.05 | 0.28 |
| I_public gathering restrictions | -0.12 | 0.05 | 0.02 | 0.50 | 0.03 | <0.01 |
| I_ closure of public spaces | 1.46 | 0.42 | <0.01 | -0.35 | 0.04 | <0.01 |

* Scenario 2: ≥50 RSV tests per week, model without seasonality (week variable) and weeks with RSV rate=0 deleted.

** Year describes the variation between years for countries; week describes the variation between weeks within a year (polynomial); measure describes the interaction between individual NPIs and RSV during the epidemiological year (defined as week 27 through week 26); I_measure describes the interaction between NPIs and RSV during weeks in which RSV activity is increasing (from the onset to peak of RSV epidemics).

Abbreviations: NPIs, non-pharmaceutical interventions; SE, standard error; RSV, respiratory syncytial virus
